# Complex context relationships between DNA methylation and accessibility, histone marks, and *hTERT* gene expression in acute promyelocytic leukemia cells: perspectives for all‐*trans* retinoic acid in cancer therapy

**DOI:** 10.1002/1878-0261.12681

**Published:** 2020-04-22

**Authors:** Delphine Garsuault, Claire Bouyer, Eric Nguyen, Rohan Kandhari, Martina Prochazkova‐Carlotti, Edith Chevret, Patricia Forgez, Evelyne Ségal‐Bendirdjian

**Affiliations:** ^1^ Team: Cellular Homeostasis, Cancer, and Therapies INSERM UMR‐S 1124 Université de Paris France; ^2^ Université de Paris Paris Sorbonne Cité France; ^3^ Paris‐Sud University Paris‐Saclay University Orsay France; ^4^ Indian Institute of Technology BHU Varanasi India; ^5^ Team Cutaneous Lymphoma Oncogenesis INSERM U1053 Bordeaux France; ^6^ BioMedTech Facilities, CNRS UMS2009/INSERM US36 Université de Paris France

**Keywords:** acute promyelocytic leukemia, ATRA, DNA methylation, histone marks, *hTERT* promoter, telomerase

## Abstract

Telomerase (hTERT) reactivation and sustained expression is a key event in the process of cellular transformation. Therefore, the identification of the mechanisms regulating *hTERT* expression is of great interest for the development of new anticancer therapies. Although the epigenetic state of *hTERT* gene promoter is important, we still lack a clear understanding of the mechanisms by which epigenetic changes affect *hTERT* expression. Retinoids are well‐known inducers of granulocytic maturation in acute promyelocytic leukemia (APL). We have previously shown that retinoids repressed *hTERT* expression in the absence of maturation leading to growth arrest and cell death. Exploring the mechanisms of this repression, we showed that transcription factor binding was dependent on the epigenetic status of *hTERT* promoter. In the present study, we used APL cells lines and publicly available datasets from APL patients to further investigate the integrated epigenetic events that promote *hTERT* promoter transition from its silent to its active state, and inversely. We showed, in APL patients, that the methylation of the distal domain of *hTERT* core promoter was altered and correlated with the outcome of the disease. Further studies combining complementary approaches carried out on APL cell lines highlighted the significance of a domain outside the minimal promoter, localized around 5 kb upstream from the transcription start site, in activating *hTERT.* This domain is characterized by DNA hypomethylation and H3K4Me3 deposition. Our findings suggest a cooperative interplay between *hTERT* promoter methylation, chromatin accessibility, and histone modifications that force the revisiting of previously proposed concepts regarding *hTERT* epigenetic regulation. They represent, therefore, a major advance in predicting sensitivity to retinoid‐induced *hTERT* repression and, more generally, in the potential development of therapies targeting *hTERT* expression in cancers.

## Graphical abstract

Telomerase (hTERT) reactivation is a key event in oncogenesis. Although the epigenetic‐based regulation of hTERT is important, we still lack a clear understanding of the mechanisms by which epigenetic changes affect *hTERT* gene promoter. Our findings suggest a cooperative interplay between *hTERT* promoter methylation, chromatin accessibility, and histone modifications that force the revisiting of previously proposed concepts regarding *hTERT* regulation.

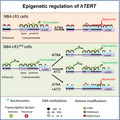

**Abbreviations**
APL: acute promyelocytic leukemia
ATO: arsenic trioxide
ATRA: all‐*trans* retinoic acid
BAC: bacterial artificial chromosome
FISH: fluorescence *in situ* hybridization
GAPDH: glyceraldehyde‐3‐phosphate dehydrogenase
hTERT: human telomerase reverse transcriptase
hTR: human telomerase RNA
NOMe‐seq: nucleosome occupancy and methylome sequencing
qRT‐PCR: quantitative reverse transcriptase polymerase chain reaction
RRBS: reduced representation bisulfite sequencing
SAM: *S*‐adenosylmethionine
SNP: single nucleotide polymorphism
SSC: saline sodium citrate
TPE‐OLD: telomere position effect over long distances
TSS: transcription start site

## 1 Introduction

Telomere maintenance is a primary and universal characteristic of cellular transformation, leading to unlimited replicative capacity. Without telomere maintenance, the other hallmarks of cancer described by Hanahan and Weinberg (2011) could neither persist nor contribute to the subsequent cancerous events. This maintenance is performed mainly by a specialized ribonucleoprotein complex, the telomerase. Any strategy to block telomerase activity or expression specifically in tumor cells, to force them to enter replicative senescence or apoptosis, may contribute to innovative therapeutic developments. Up to now, strategies targeting telomerase activity inhibition have been disappointing because of low efficacy and long‐term toxicity of the drugs. These failures are partially due to an insufficient understanding of telomerase regulation mechanisms.

The human telomerase complex consists of a catalytic reverse transcriptase protein subunit (hTERT) encoded by *TERT* gene located on chromosome 5 (5p15.33) (Meyerson *et al.*, 1997), an RNA template (*hTR*) encoded by *TERC* gene located on chromosome 3 (3q26.2) (Shippen‐Lentz and Blackburn, 1990) and accessory proteins required for proper telomerase assembly and recruitment to chromosomes (Cohen *et al.*, 2007).

*hTERT* expression is the primary determinant and the limiting factor for telomerase activity. The regulation of *hTERT* expression in human cancers is consequently of major importance. *hTERT* expression is tightly regulated at the transcriptional level (Avilion *et al.*, 1996). As the reactivation of *hTERT* is critical in carcinogenesis and tumor progression, it is essential to further advance in our understanding of *hTERT* regulation at the transcriptional level. Several transcription factors, either repressors (such as Mad1, E2F, WT1, and MZF2) or activators (such as c‐Myc, NF‐kB, and Sp1) are important in the tight control of *hTERT* expression (Ramlee *et al.*, 2016; Renaud *et al.*, 2005). However, these factors are involved in the regulation of numerous normal cells and thereby are difficult to be specifically targeted in cancer cells. Recent studies have identified cancer‐associated *hTERT* promoter mutations as a genetic mechanism for *hTERT* upregulation. The most frequent mutations are found upstream of the transcription start site (TSS) at 1 295 228 (C288T), and 1 295 250 (C250T). These mutations generate novel binding sites for the ETS (E26 transformation‐specific or E‐twenty‐six) transcription factors and thereby, alter positively the transcriptional regulation of *hTERT* (Bell *et al.*, 2015; Horn *et al.*, 2013; Huang *et al.*, 2013; Vinagre *et al.*, 2013). Besides, *hTERT* upregulation occurs in the absence of these promoter mutations in many tumor types, suggesting that other mechanisms are involved, and in particular, epigenetic mechanisms. Indeed, the epigenetic state of *hTERT* promoter is important for the tight control of *hTERT* expression. However, despite extensive studies on *hTERT* promoter DNA methylation alteration, contradicting results have been reported in the literature (Azouz *et al.*, 2010; Dessain *et al.*, 2000; Devereux *et al.*, 1999; Guilleret and Benhattar, 2003; Guilleret *et al.*, 2002; Losi *et al.*, 2019; Zinn *et al.*, 2007). Therefore, we still lack a clear understanding of the underlying mechanisms by which epigenetic changes affect *hTERT* expression, and if they can be specifically targeted.

DNA methylation is often linked to histone post‐translational modifications (Bannister and Kouzarides, 2011) that affect the compaction state of chromatin, and thereby gene expression by controlling the accessibility of transcription factors to the promoter. Nucleosomes have classically been thought to prevent DNA sequence from interacting with transcription factors (either activators or repressors). Therefore, the degree of nucleosome occupancy along DNA in the chromatin contributes significantly in the activation and repression of chromatin regions because it modulates the accessibility of DNA to the transcriptional machinery and regulatory proteins (Li *et al.*, 2007). Many factors have been proposed to directly regulate the nucleosome positioning (Lai and Pugh, 2017), including the genomic sequence, DNA methylation (Chodavarapu *et al.*, 2010), and histone modifications. Importantly, the higher‐order chromatin organization results in the formation of looping structures that could play a significant role in gene activity by clustering genomic loci into domains via long‐range interactions (Rowley and Corces, 2018). This chromatin looping is also influenced by nucleosome positioning and therefore by epigenetic modifications. The tight linkage between *hTERT* expression and DNA methylation, histone marks, chromatin accessibility and subnuclear localization of *hTERT* has not been previously formally examined and was addressed in this study.

All‐*trans* retinoic acid (ATRA) is widely used as first‐line therapy in acute promyelocytic leukemia (APL) as an inducer of granulocytic maturation of APL blasts. Besides, we have previously reported that long‐term ATRA treatment could induce telomere‐dependent cell death in some ATRA‐maturation‐resistant cells. Indeed, in the maturation‐resistant NB4‐LR1 cells, pharmacological concentrations of ATRA induced strong repression of *hTERT* leading to telomere shortening and cell death (Pendino *et al.*, 2003, 2001, 2006, 2003, 2001, 2006, 2003, 2001, 2006). This observation suggests that ATRA, by targeting *hTERT*, can exert antitumoral properties independently of its action on differentiation. A variant of the NB4‐LR1 cell line, named NB4‐LR1^SFD^, resistant to telomerase‐dependent ATRA‐induced cell death was selected. The NB4‐LR1^SFD^ cells are characterized by a steady expression of *hTERT* despite the continuous presence of ATRA (Pendino *et al.*, 2002). Understanding the mechanisms of ATRA‐induced *hTERT* repression will lead to the development of new therapeutical strategies to improve ATRA responsiveness. Thus, the two ATRA‐maturation‐resistant APL cell sublines described above, which behave distinctly to long‐term ATRA treatment regarding the influence on *hTERT* expression, are a valuable model to study the molecular events leading to *hTERT* repression and reactivation in cancer. We have previously shown that transcription factor binding to *hTERT* promoter is dependent on the epigenetic status of the promoter, including DNA methylation (Azouz *et al.*, 2010). In the present study, we took advantage of the two well‐established cell lines, in which *hTERT* expression and telomerase activity are oppositely regulated by retinoids to explore in more details the interplay between *hTERT* gene positioning in the nucleus, DNA methylation, nucleosome occupancy, and histone modifications at *hTERT* promoter. To this end, our investigation combines a highly sensitive single‐molecule nucleosome occupancy and methylome sequencing assay (NOMe‐seq) with histone ChIP analysis and 3D‐FISH to generate an integrated view of chromatin organization and gene expression at the level of *hTERT*.

## 2 Materials and methods

### 2.1 Patients

DNA methylation data from bone marrow samples of 18 APL patients at diagnosis, eight matched patient at remission, one sample from an APL patient, treated or not *ex vivo* with ATRA for 48 h, CD34^+^ cells from eight healthy donors, and promyelocytes generated *in vitro* from these CD34 cells, were downloaded from the GEO website (https://www.ncbi.nlm.nih.gov/gds/?term=GSE42119%5BAccession%5D) (Schoofs *et al.*, 2013). Histone modifications from three APL patients (pz‐302, a non‐high‐risk primary APL patient and pz‐284 and pz‐289, two high‐risk primary APL patients resistant to standard ATRA plus chemo treatment) were downloaded from the BLUEPRINT data portal (http://dcc.blueprint-epigenome.eu/#/experiment) and visualized in UCSC genome browser (https://genome.ucsc.edu/).

### 2.2 Chemicals, cell lines, cell culture, and treatments

All*‐trans* retinoic acid (ATRA), arsenic trioxide (ATO), and protease inhibitor cocktail (P8340) were purchased from Sigma (St Louis, MO, USA). The maturation‐resistant human APL cell lines, NB4‐LR1 and NB4‐LR1^SFD^, were cultured as previously described (Pendino *et al.*, 2001, 2002, 2001, 2002). All cells were cultured at 37 °C in a humidified incubator with 5% CO_2_ (Binder Incubators, Nanterre, France). For treatments, cells were seeded in medium containing 1 µm of ATRA, 0.2 µm of ATO alone or in combination.

### 2.3 Quantitative reverse transcriptase polymerase chain reaction (qRT‐PCR)

Total cellular RNA was extracted using TRIzol reagent (Invitrogen, Thermo Fisher Scientific, Courtaboeuf, France) according to the manufacturer's instructions and subjected to reverse transcriptase reaction with oligo(dT) using Transcriptor First Strand cDNA Synthesis kit (Roche Diagnostics, Meylan, France) as described in the manufacturer's instructions. The cDNAs were subsequently submitted to quantitative real‐time PCR using the LightCycler technology and the Light Cycler FastStart DNA MasterPLUS SYBR Green Kit (Roche Diagnostics). *hTERT* levels were normalized to the expression of glyceraldehyde‐3‐phosphate dehydrogenase (*GAPDH*) used as the internal control gene. Primer sequences and their localization on the *hTERT* gene are shown in Table S1 and Fig. S1.

### 2.4 Sanger sequencing

The presence of *hTERT* promoter/enhancer mutations was evaluated by conventional Sanger sequencing. Genomic DNA was extracted from cells as previously reported (Ségal‐Bendirdjian and Jacquemin‐Sablon, 1995). *hTERT* core promoter (region I: from the position −650 to +150 bp relative to the TSS) and a distal regulatory upstream region (region II: from the position −5500 to −4900 bp relative to the TSS) were amplified using specific primers whose sequences and localizations are reported in Table S1 and Fig. S1.

### 2.5 Nucleosome occupancy and methylome sequencing

Nucleosome occupancy and methylome sequencing was performed as previously described (Kelly *et al.*, 2012). In brief, nuclei were isolated, resuspended in 165 µL ice‐cold GpC buffer [1× M.CviPI GC buffer, 0.3 m sucrose, 160 µm *S*‐adenosylmethionine (SAM)], and split into two aliquots. One aliquot was treated with 75 U of GpC methyltransferase M.CviPI, the other was incubated with the same amount of water. The tubes were incubated for 15 min at 37 °C before adding a boost of 75 U of M.CviPI, and 160 µm SAM in the treated nuclei for an additional 15 min at 37 °C. The reaction was terminated by the addition of one volume of stop solution (20 nm Tris/HCl pH 8, 600 mm NaCl, 1% SDS, 10 mm EDTA, 400 µg·mL^−1^ proteinase K) and the samples were incubated for 2 h at 55 °C. DNA was then purified by phenol/chloroform extraction and ethanol precipitation. Bisulfite conversion was performed on 1 µg of purified DNA using the EZ DNA Methylation Kit (Zymo Research, Ozyme, SAS, Saint‐Cyr‐L'Ecole, France). Bisulfite‐converted DNA was used for PCR amplification of the regions of interest with the primers reported in Table S1. We designed NOMe‐seq assays to explore region I and II as defined above (see Fig. S1). PCR amplicons were purified with NucleoSpin Gel and PCR Clean‐up kit (Macherey‐Nagel, Hoerdt, France) and cloned into the pGEM‐T Easy vector (Promega, Charbonnières‐les‐Bains, France) as described in the manufacturer's instructions. For each experiment, 10–20 plasmid subclones were sequenced (MWG Biotech, Ebersberg, Germany) for the assessment of nucleosome occupancy and CpG methylation. The M.CviPI enzyme methylates GpC sites in accessible DNA, whereas nucleosome bound DNA, which is inaccessible, remains refractory to GpC methylation. Reactions without M.CviPI were routinely performed to confirm endogenous CpG methylation levels. Besides, NOMe‐seq retains the endogenous methylation status of the DNA allowing nucleosome positions and DNA methylation to be determined on the same molecule. The efficiency of M.CviPI GpC methyltransferase was high (95%) and the bisulfite conversion rate was 99% on average. The methylation patterns of the individual clones are presented in Fig. S2. The data visualization as methylation profiling plot was performed using Methylation plotter web tool (Mallona *et al.*, 2014).

### 2.6 Chromatin immunoprecipitation

ChIP assay was performed using Magna ChIP kit (Merck Millipore, Guyancourt, France), following the manufacturer's instructions. Briefly, 10^7^ cells were cross‐linked with 1% paraformaldehyde and sonicated to obtain fragments ranging from 300 to 600 bp (Bioruptor Pico; Diagenode Diagnostics, Seraing, Belgium). An aliquot (3 µL, 6000 cells) of the sonicated chromatin was used as input fraction to quantify the total amount of DNA. For immunoprecipitation, 4 µg of antibodies were prebound to 20 µL protein A/G magnetic beads and incubated with chromatin overnight at 4 °C. As a negative control, IgG of the same species as the antibody of interest was included. The following antibodies were used for the immunoprecipitation: H3K27Me3 (Merck Millipore, #07‐449); H3K4Me3 (Merck Millipore, #04‐745); H3Ac (Merck Millipore, #06‐599), H3K9Me3 (Merck Millipore, #07‐442); IgG (Merck Millipore, #PP64B). Three biological replicates were produced independently at a different time of cell culture. Immunoprecipitated chromatin samples were un‐cross‐linked and purified. Immunoprecipitated and input DNA were then analyzed by quantitative polymerase chain reaction (qPCR, LightCycler 2.0; Roche) with the appropriate primers targeting regions upstream and downstream *hTERT* TSS (Fig. S1). The primer sequences are indicated in Table S1. The amount of immunoprecipitated DNA is represented as a normalized signal to total input DNA used in each immunoprecipitation.

### 2.7 3D fluorescence *in situ* hybridization

Fluorescence *in situ* hybridization assay was performed as previously described (Chaumeil *et al.*, 2013). Briefly, 2 × 10^6^ cells were fixed with 2% paraformaldehyde and spread on Superfrost slides (Menzel‐Gläzer, Fisher Scientific, Illkirch, France) before permeabilization with 0.5% Triton in PBS. Slides were dehydrated with a gradual concentration of ethanol (70%, 85%, and 100%) and then treated with RNase A (100 µg·mL^−1^) for 1 h at 37 °C (Neobrite, NB12‐0001; NeoBiotech, Rotterdam, Netherlands). A second permeabilization step with 0.7% Triton in 0.1 m HCl was performed before denaturation with 50% formamide in 2× SSC (saline sodium citrate) solution at 80 °C for 30 min. Probes were denatured at 75 °C for 5 min and added to the sample for hybridization overnight at 37 °C. After 3 washes in 2× SSC/50% formamide, three washes in 2× SSC, one wash in 0.5× SSC and one wash in PBS, slides were incubated with Hoechst 33342 (1 µg·mL^−1^ in PBS) for 15 min at room temperature and mounted with Mowiol. Probes were prepared using nick translation kit (Vysis kit; Abbott Laboratories, Rungis, France) following the manufacturer's instructions. Bacterial artificial chromosome (BAC) plasmids were purchased from Source BioScience (Biovalley SA, Illkirch, France: RP11‐117B23 for *hTERT* locus staining (Spectrum Orange, 552 nm), RP11‐44H14 for subtelomeric region 5p staining (Spectrum Green, 496 nm), and RP11‐846K3 for staining ‘intermediary’ region, containing *TPPP* and *CEP72* genes (Spectrum Green, 496 nm). The positions of the FISH probes are indicated in Fig. S2. Labeled probes were precipitated by adding 10‐fold excess of Cot‐1 DNA, 1/10 volume of 3 m sodium acetate (NaAc, pH 5.2) and 3 volumes of 100% ethanol. Probes were then centrifuged at 12 000 ***g*** for 45 min at 4 °C. After washing with 70% ethanol, probes were then dried at 37 °C for 10 min and resuspended in 50 µL of hybridization buffer (2× SSC/50% formamide/10% dextran sulfate). Images were acquired using LSM 710 confocal microscope (Carl Zeiss, Marly le Roi, France). A 63× Plan‐Apochromat oil immersion objective was used to capture optical sections at intervals of 0.37 µm. LSM‐type images were generated and processed with imagej (https://imagej.nih.gov/ij/) and JACoP plugin. The 3D distance between each center of the deconvolved fluorescent spot of *hTERT* locus, and either the subtelomeric 5p region or the intermediate locus were collected and analyzed with graphpad prism (San Diego, CA, USA).

### 2.8 Statistical analysis

Statistical analysis was conducted using graphpad prism 6.01 software. The difference between groups was analyzed using unpaired or paired Student's *t*‐test when there were only two groups or assessed by one‐way ANOVA followed by Tukey's multiple comparison tests when there were more than two groups. All tests carried out were two‐tailed. Differences were considered as significant when *P* < 0.05.

## 3 Results

### 3.1 Identification of an *hTERT* promoter methylation signature in APL patient samples

**Fig. 1:**
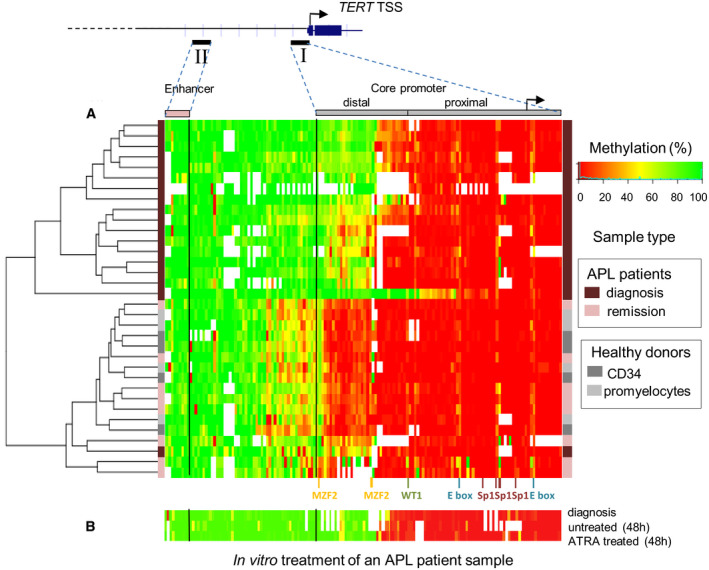
Hierarchic cluster analysis of APL patients' samples and methylation profile at *hTERT* promoter and enhancer. (A) The methylation heat map generated from the unsupervised hierarchical clustering is based on raw RRBS (reduced representation bisulfite sequencing) DNA methylation values in patients at diagnosis and remission, and healthy donors. (B) *In vitro* ATRA treatment of one APL patient sample for 48 h. White color indicates unavailability of data. The color grid on each side visualizes the sample characteristics.

In a previous work performed on APL cell lines, DNA methylation analysis of the *hTERT* promoter led us to identify two distinct functional domains differentially methylated, a proximal one fully unmethylated and a distal one whose methylation modifications could account for the capacity of ATRA to repress *hTERT* gene (Azouz *et al.*, 2010). To evaluate the clinical relevance of this particular epigenetic pattern, we analyzed the methylation profile of *hTERT* promoter up to −5 kb upstream the TSS in eighteen patient samples at primary diagnosis using a publicly available APL dataset (Schoofs *et al.*, 2013) (http://www.ncbi.nlm.nih.gov/geo/query/acc.cgi?acc=GSE42119). Bone marrow samples of eight matched patients in remission were also analyzed. CD34^+^ cells from healthy donors and promyelocytes generated *in vitro* from these CD34^+^ cells were included in the analysis as controls. Raw methylation levels from CpG sites covered by RRBS across all samples were used to perform unsupervised hierarchical clustering (Fig. 1A). The samples formed two main clusters: one (top) encompassing APL patient samples at diagnosis and one (bottom) encompassing bone marrow samples of patients in remission and hematopoietic progenitor cells from healthy donors. In patients at diagnosis, we identified a region close to the TSS that was largely hypomethylated, while the distal region further upstream (−200 bp upstream of the TSS) was significantly more methylated. The dual methylation pattern, already reported in APL cell lines (Azouz *et al.*, 2010), was therefore confirmed in APL patient samples at diagnosis. Interestingly, the global methylation level of the distal region of the core promoter differs and this difference clusters with the patient conditions. Indeed, the methylation of the CpG sites within the distal region was significantly lower in patients at remission and in healthy donors compared to patients at diagnosis. Only one patient at diagnosis clusters with the remission patients. It is worth noting that *in vitro* ATRA treatment of primary APL cells from one APL patient for 48 h did not change significantly the pattern of DNA methylation of *hTERT* promoter (Fig. 1B). One potential explanation for this might be that a 48‐h ATRA treatment of APL cells *in vitro* could not completely mimic conditions of ATRA treatment of patients. Of note, no expression data of *hTERT* relative to the dataset analyzed were available neither for APL patients at diagnosis nor for patients at remission. This rules out the possibility of performing any correlation between the methylation pattern of *hTERT* gene promoter and its expression.

Nevertheless, these results underline the significance of the epigenetic modification of this distal region in *hTERT* expression regulation, and indicate that the DNA methylation pattern of this region can represent a potential indicator for the diagnosis and the monitoring of APL disease outcome.

### 3.2 NB4‐LR1 and NB4‐LR1^SFD^, two ATRA‐maturation‐resistant APL cell lines, a tool to investigate the epigenetic regulation of the *hTERT* gene

**Fig. 2:**
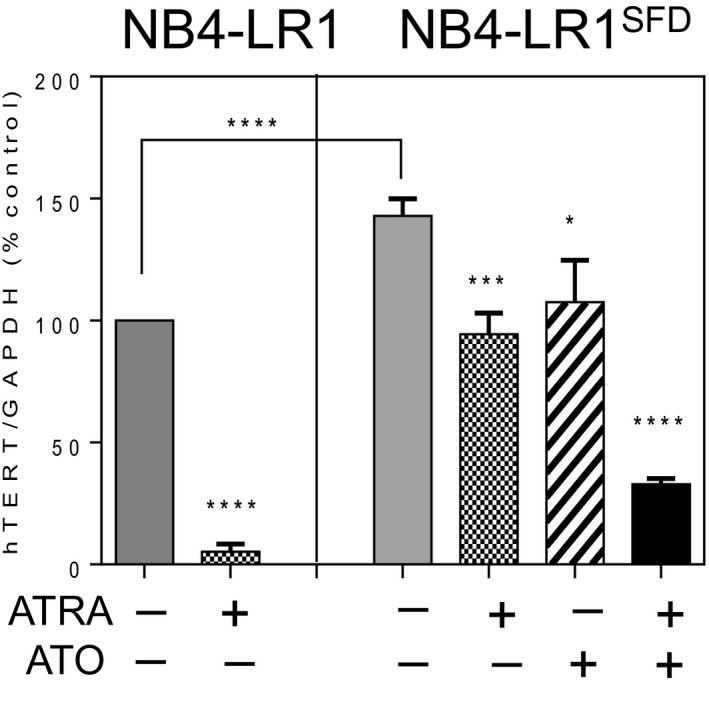
*hTERT* mRNA expression in ATRA‐maturation‐resistant APL cell lines. Cells were treated for 7 days with ATRA (1 µm) alone or in combination with ATO (0.2 µm). *hTERT* gene expression levels in NB4‐LR1 and NB4‐LR1^SFD^ cells were measured by qRT‐PCR. The levels were normalized to GAPDH expression and the results were expressed as a percentage of that detected in untreated NB4‐LR1 cells (±SEM). *t*‐Test or one‐way ANOVA with *post hoc* Tukey, *P < 0.05, ***P < 0.001, ****P < 0.0001.

The results obtained in APL patients encouraged us to perform a more comprehensive analysis of the epigenetic status of *hTERT* gene promoter. To carry out this study, we took advantage of two well‐established APL cell lines, in which *hTERT* expression and telomerase activity are regulated by retinoids in an opposite way. The NB4‐LR1 cell line, derived from an APL patient, is resistant to ATRA‐induced maturation (Duprez *et al.*, 1992; Lanotte *et al.*, 1991). In this cell line, as previously reported, the long‐term treatment with ATRA induced a strong repression of *hTERT* (Fig. 2). In the NB4‐LR1^SFD^ cell line, established from the NB4‐LR1 cells, *hTERT* expression has been stably reactivated (Pendino *et al.*, 2001, 2002, 2001, 2002). In this cell line, the constitutive expression of *hTERT* is higher than in the NB4‐LR1 cell line and remained high despite ATRA treatment. *hTERT* repression is, however, achieved when ATRA treatment was combined with ATO (Tarkanyi *et al.*, 2005). Therefore, these two specific cell lines are illustrative of tumor progression process, and they represent excellent tools to understand how tumor cells can install a finely tuned transcriptional regulation for *hTERT* and bypass its repression. One mechanism that can explain, at least partly, the reactivation of *hTERT* is mutations at specific loci of the *hTERT* promoter (Vinagre *et al.*, 2013). *hTERT* promoter sequencing showed that neither NB4‐LR1 nor NB4‐LR1^SFD^ cells harbored the recurrent promoter mutations known to generate ETS binding sites at −124 bp (C228T) and −146 bp (C250T) upstream of the start codon (Bell *et al.*, 2015; Horn *et al.*, 2013; Huang *et al.*, 2013; Vinagre *et al.*, 2013). Sequence analysis revealed, the presence of already known single nucleotide polymorphisms (SNPs) in *hTERT* core promoter and a region approximately 5 kb upstream of the *hTERT* TSS previously described as an enhancer element (Eldholm *et al.*, 2014) (Fig. S1 and Table S2). As the SNP observed were found in both cell lines, they could not explain the distinct responses of NB4‐LR1 and NB4‐LR1^SFD^ cells to long‐term ATRA treatment regarding *hTERT* expression, indicating that other mechanisms may be involved, most likely epigenetic mechanisms. Therefore, the above cell lines would serve as valuable cell models to investigate epigenetic events involved in telomerase regulation.

### 3.3 *hTERT* promoter DNA methylation and nucleosome occupancy by NOMe‐seq in ATRA‐maturation‐resistant APL cell lines

**Fig. 3:**
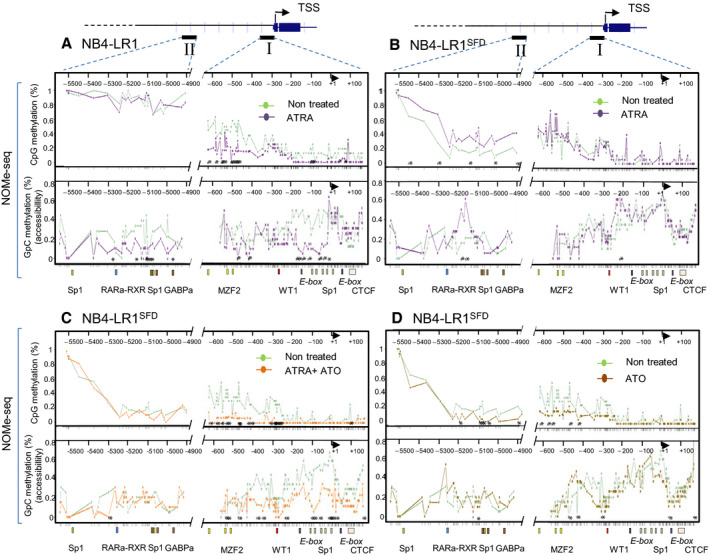
Chromatin accessibility and endogenous CpG methylation at the *hTERT* gene promoter as determined by NOMe‐seq analysis. NOMe‐seq was used to determine in the same time the level of endogenous DNA methylation and chromatin accessibility on individual DNA molecules for two subregions of the *hTERT* gene promoter visualized on the upper part of the figure as region I and region II. Region I encompasses *hTERT* TSS, and region II corresponds to the upstream conserved sequence described as an enhancer. For each sequence, 10–20 clones were analyzed from DNA obtained from NB4‐LR1 (panel A) and NB4‐LR1^SFD^ (panel B–D) cells treated or not as indicated. The upper part of each panel indicates endogenous DNA methylation, the lower part chromatin accessibility. Data visualization as methylation profiling plot was performed using Methylation plotter web tool (Mallona *et al.*, 2014). Each line represents for each group of samples the methylation mean for each position. Asterisks indicate a statistical significance between the treated and untreated groups as calculated by Kruskal–Wallis test (*P* < 0.05). Ticks in *x*‐axis indicate individual CpG (upper panel) and GpC (lower panel), respectively. Transcription factor binding sites (colored boxes) and TSS (solid arrow) are depicted.

**Fig. 4:**
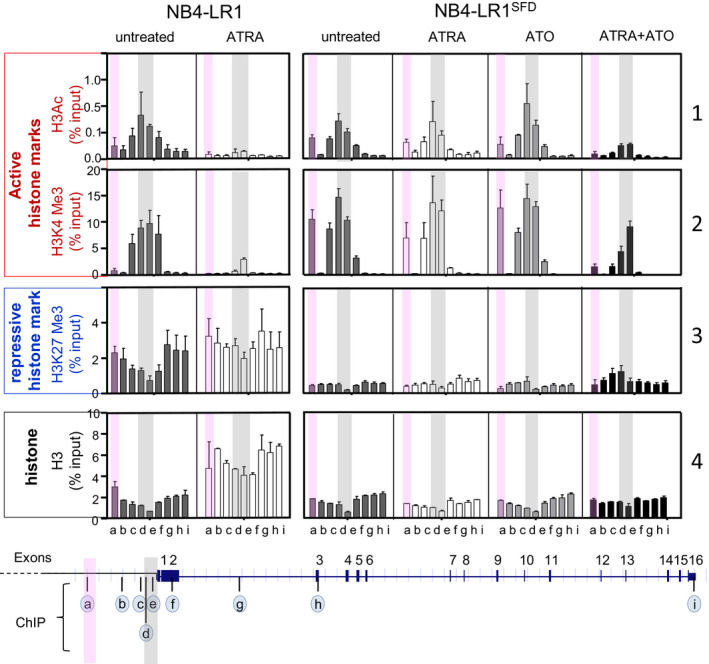
Chromatin marks at *hTERT* gene. ChIP was analyzed by quantitative PCR (qPCR) using nine pairs of primers to amplify selected regions (a to i) encompassing the *hTERT* gene as indicated on the schematic representation above the graphs. Semitransparent gray and pink color labels the analyzed regions of *hTERT* proximal promoter and enhancer, respectively. Site‐specific ChIPs of H3Ac, H3K4Me3, H3K27Me3, and H3 are presented as percentage of the input ± the SEM of three independent experiments.

To investigate the relationship between DNA methylation and nucleosome occupancy in ATRA‐treated and untreated NB4‐LR1 and NB4‐LR1^SFD^ cells and resolve the pattern of methylation at *hTERT* promoter gene, we applied a high‐resolution, single‐molecule analysis named NOMe‐seq. This procedure allows the simultaneous investigation of nucleosome occupancy and endogenous CpG methylation on the same DNA molecule and the analysis of the relationships between these two chromatin features on a single locus (Kelly *et al.*, 2012). We focused on two different regions of *hTERT* promoter: the first region (region I) extended from −650 to +150 bp relative to TSS; the second region (region II) located far upstream from the TSS (−5500 to −4900 bp) (Eldholm *et al.*, 2014) identified as a putative enhancer domain (Fig. 3, Figs [Link], [Link]). In region I, *hTERT* promoter DNA was weakly methylated with a mean methylation level of 23.7% and 14.7% in untreated NB4‐LR1 and NB4‐LR1^SFD^ cells, respectively. We confirmed the dual methylation pattern observed in APL patients, as the region close to the TSS is largely hypomethylated in both cell lines, while the region further upstream (about −600 to −200 bp upstream of the TSS) was significantly more methylated (Zinn *et al.*, 2007). In NB4‐LR1 cells, ATRA‐induced repression of *hTERT* was associated with a global decrease of CpG methylation level in the region I (from 23.7% to 9.4%), suggesting that the methylation status of the proximal promoter may contribute to *hTERT* gene silencing. Associated with this decrease of DNA methylation, we observed a striking loss of chromatin accessibility particularly in the region between −200 and +1 bp. The size of this region is large enough to accommodate at least one nucleosome. This observation was supported by the enrichment of histone H3 observed in this region in the ChIP‐qPCR assay (Fig. 4, panel 4, amplicon e). Similarly, in NB4‐LR1^SFD^ cells, the important repression of *hTERT* after treatment with ATRA and ATO in combination was associated with a hypomethylation of region I (from 14.7% to 2.2%) and a global reduction in chromatin accessibility. Of note, ATRA treatment alone did not affect either CpG methylation or chromatin accessibility in those cells. In NB4‐LR1^SFD^ cells, ATO treatment alone induced a decrease of DNA methylation only in the distal part of *hTERT* core promoter without major modifications in chromatin accessibility.

In region II, NB4‐LR1 cells displayed a marked global hypermethylation (89.1%). Despite no significant change in the DNA methylation profile of this region was observed in ATRA‐treated NB4‐LR1 cells, a decrease of chromatin accessibility was noticed. In NB4‐LR1^SFD^ cells, this region displayed strikingly a variable but globally lower methylation levels (33.5%) compared to that in NB4‐LR1 cells. An increase of the DNA methylation associated with a partial increase in chromatin accessibility is observed after ATRA treatment of NB4‐LR1^SFD^ cells. However, neither DNA methylation nor chromatin accessibility was modified after treatment with ATRA and ATO alone or in combination.

Altogether, these results indicate that changes in the methylation status of *hTERT* promoter are probably a necessary but not a sufficient condition for an efficient transcriptional repression of this gene; they need to be associated with a decrease in chromatin accessibility.

### 3.4 Histone marks at *hTERT* promoter in ATRA‐maturation‐resistant APL cell lines and patients

The histone modifications have been reported to play a significant role in the regulation of gene expression, including *hTERT* (Cong and Bacchetti, 2000; Lewis and Tollefsbol, 2016; Won *et al.*, 2002). Therefore, we conducted site‐specific ChIP‐qPCR assays to examine the relationship between *hTERT* expression and histone marks at specific positions along the *hTERT* gene. Antibodies specific to one repressive (H3 trimethylated lysine 27, H3K27Me3), two active (H3 trimethylated lysine 4, H3K4Me3, and acetylated lysine H3, H3Ac) marks, and the total histone H3 were used for ChIP assay (Fig. 4).

In both untreated cell lines, the active marks, H3K4Me3 and H3Ac (Fig. 4, panel 1 and 2), were enriched at *hTERT* core promoter (region in gray mapped by primer sets *d*, and *e*), indicating a permissive transcriptional status of the chromatin. In NB4‐LR1 untreated cells, the active H3K4Me3 mark (Fig. 4, panel 2) was included within a larger region of H3K27Me3 repressive mark (Fig. 4, panel 3). This concurrence of H3K4Me3 and H3K27Me3 across the *hTERT* promoter and TSS was already described (Zinn *et al.*, 2007). This epigenetic feature is a characteristic of bivalent domains (or ‘poised’ promoters) (Bernstein *et al.*, 2006), suggesting an effective plasticity of the chromatin at this location of the *hTERT* promoter, crucial for the transition between active and repressive states. This feature was not observed in NB4‐LR1^SFD^ cells since H3K27Me3 remained very low across the region of the *hTERT* gene probed by the nine primer sets (Fig. 4, panel 3). Remarkably, in the region mapped by the primer set *a* (region in pink), H3K4Me3 and to a lesser extent H3Ac, were significantly enriched in NB4‐LR1^SFD^ cells as compared to NB4‐LR1 cells (Fig. 4, panel 2).

After a 7‐day ATRA treatment, the levels of these active marks decreased dramatically only in NB4‐LR1 cells and remained unchanged in NB4‐LR1^SFD^ cells. Nevertheless, their presence decreased in NB4‐LR1^SFD^ exposed to the combination of ATRA and ATO. The decrease occurred at the *hTERT* core promoter (region mapped by primers *c*, *d*, and *e*) as well as in the region mapped by the primer set *a*.

In NB4‐LR1 cells, the repressive H3K27Me3 mark was enriched upstream and downstream of the *hTERT* core promoter regions (Fig. 4, panel 3). Its level further increased after ATRA treatment. In contrast, in NB4‐LR1^SFD^ cells, the level of this mark remained rather weak, even though a modest increase was observed when treated with ATRA and ATO in combination. Histone H3 pull‐down was used to map the underlying distribution of histones ChIP experiments using anti‐histone H3 to map the distribution of histones revealed a higher level of histone H3 in ATRA‐treated NB4‐LR1 cells than in untreated ones (Fig. 4, panel 4). Such a difference was not readily observable in NB4‐LR1^SFD^ cells. As mentioned above, this increase is likely to reflect a change in nucleosome positioning in this region of the *hTERT* gene (Fig. 3).

Altogether, these results confirm that changes in *hTERT* expression are associated with changes in the pattern of histone post‐translational modifications. Furthermore, and importantly, they identify a new *hTERT* gene region located at about −5 kb upstream of the TSS enriched for active H3K4Me3 mark or H3K27Me3 in NB4‐LR1^SFD^ and NB4‐LR1 cells, respectively. The loss of the loss of the H3K4Me3 marks is correlated to *hTERT* repression.

Next we used publicly available data from three APL patients to investigate whether the primary blasts from these patients present a pattern of histone modifications similar to NB4‐LR1 or NB4‐LR1^SFD^ cell lines. We observed the co‐occurrence at *hTERT* promoter of the two H3K4Me3 and H3K27Me3 marks as in NB4‐LR1 cells (Fig. S4). In addition, our analyses showed that the putative *hTERT* enhancer region is marked by the presence of either H3K4Me3 active marks or H3K27Me3 repressive marks in patients pz‐289 and pz‐302, respectively, these histone marks being mutually exclusive. Of note, this pattern of histone modifications did not change upon *in vitro* ATRA treatment. This absence of effect compared to that observed in the APL cell lines can possibly result from the short duration of the treatment, only for 24 h, compared to the 7‐day treatment of APL cell lines.

### 3.5 Analysis of the spatial genome organization at *hTERT* locus by 3D‐FISH

**Fig. 5:**
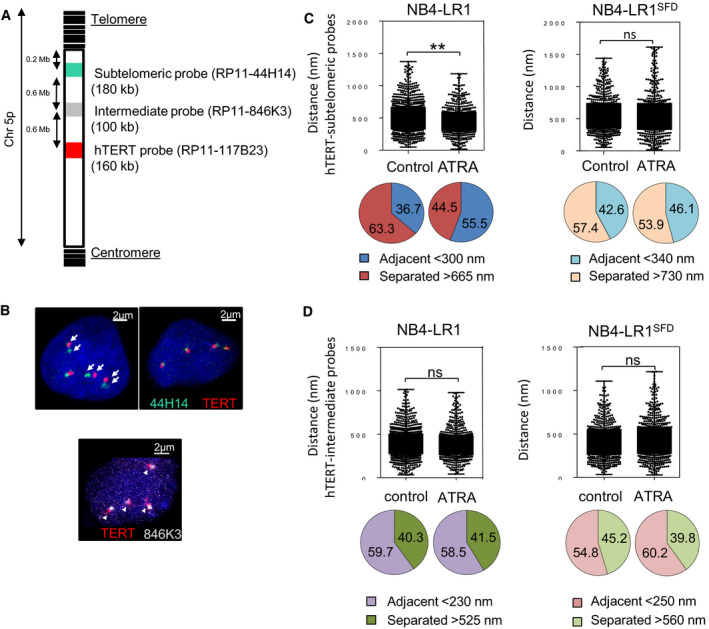
3D DNA‐FISH analysis. (A) The location of each probe is shown in a schematic representation of the short arm (p) of chromosome 5. DNA probes (RP11‐117B23 BAC) against *hTERT* locus (red) and targeting subtelomeric 5p region (RP11‐44H14 BAC, green) are used to analyze physical proximity by 3D‐FISH. (B) Confocal images of 3D‐FISH after imagej processing. Representative confocal images of 3D‐FISH showing either the proximity or the separation of the loci. DNA was counterstained with Hoechst. Arrow heads indicate spots that are considered adjacent; full arrows indicate spots that are separated. (C, D) Box plots (upper panels) illustrate the distribution of the distances determined from processed images of *in situ* hybridization using either the subtelomeric (C) or the intermediate probe (D) relative to the *hTERT* probe in NB4‐LR1 variants before and after ATRA treatment (1 µm, 7 days). Images were processed with imagej for about 150–200 nuclei from three separate experiments per condition. Significant differences are marked with asterisks (Mann–Whitney test). Pie charts (lower panels) illustrate the relative proportion of adjacent and separated signals in NB4‐LR1 and NB4‐LR1^SFD^ cells. The criteria for the analyses depending on both the probes and the cell lines are indicated. BAC probes were more adjacent in the ATRA‐treated NB4‐LR1 cells than in control cells.

The 3D genome spatial organization has been recently proposed to control nuclear structure and gene expression. Indeed, spatial chromosome arrangement can bring regulatory elements nearby the genes under their control (Dekker and Mirny, 2016). Likewise, telomeres can make looping structures and partly regulate gene expression, including the *hTERT* gene located 1.2 Mb from the end of chromosome 5p (Kim *et al.*, 2016; Robin *et al.*, 2014). This mechanism, known as telomere position effect over long distances (TPE‐OLD), would possibly influence gene expression over long distances. Based on recent observations supporting the concept that TPE‐OLD can induce *hTERT* repression (Kim and Shay, 2018), we performed 3D‐FISH on NB4‐LR1 and NB4‐LR1^SFD^ cell lines with or without ATRA treatment to evaluate whether potential modifications of spatial genome organization at the *hTERT* locus would correlate with *hTERT* expression. We used three BAC probes corresponding to each region of interest: subtelomeric and intermediate sequences, and *TERT* locus (Fig. 5A and Fig. S2). An example of the images acquired is shown in Fig. 5B. We measured the three‐dimensional distances of FISH signals between the *hTERT* locus and the 5p subtelomeric region and we discriminated pairs in *cis* (i.e., on the same chromosome 5) from those in *trans* (i.e., on different chromosomes 5). The spots in a pair present on the same chromosome 5 were then categorized into adjacent, intermediate, and separated. As previously reported, we analyzed the distributions of spots in the two extreme categories, that is, adjacent and separated (Kim *et al.*, 2016; Kim and Shay, 2018). Because NB4‐LR1 cells are slightly smaller than NB4‐LR1^SFD^ cells thresholds were adapted. Spots were classified as adjacent when the distance measured was lower than 300 or 340 nm in NB4‐LR1 and NB4‐LR1^SFD^ cells, respectively. Spots were classified as separated when the distance was greater than 665 or 730 nm, in NB4‐LR1 and NB4‐LR1^SFD^ cells, respectively (Fig. 5C). In the nucleus of control NB4‐LR1 and NB4‐LR1^SFD^ cells, the percentages of spots in close proximity (adjacent) were ~ 37% and ~ 43%, respectively. After 7 days of ATRA treatment, this percentage was significantly increased to ~ 55% in the nucleus of NB4‐LR1 cells, while the separated pairs decreased from ~ 63% to ~ 45%. In contrast, the distances between probes corresponding to these loci remained unchanged in the nucleus of ATRA‐treated NB4‐LR1^SFD^ cells as compared to untreated cells (Fig. 5C). Of note, we did not observe any differences in the distances between probes corresponding to the *hTERT* locus and the ‘intermediate’ sequences between untreated and ATRA‐treated in both NB4‐LR1 and NB4‐LR1^SFD^ cells (Fig. 5D). Overall, these results suggest that, in NB4‐LR1 cells, *hTERT* repression by ATRA treatment could be modulated by long‐range TPE‐OLD interactions at the *hTERT* locus.

## 4 Discussion

Since *hTERT* expression is upregulated in the majority of tumors, the elucidation of the mechanisms responsible for *hTERT* regulation will offer information that may be used for diagnosis and prognosis and could be translated into effective targeted cancer therapies. DNA methylation is functionally linked to several other epigenetic pathways, including post‐translational histone modifications (Okitsu and Hsieh, 2007; Weber *et al.*, 2007), nucleosome positioning (Huff and Zilberman, 2014), and nuclear localization. As these processes play an essential role in gene expression by regulating the condensation and accessibility of genomic DNA, they were investigated at the level of *hTERT* gene to obtain a comprehensive view of the epigenetic landscape of this gene and consequently its regulation. Therefore, we investigated the epigenetic landscape changes associated with ATRA‐induced *hTERT* gene repression with a unique approach combining several techniques.

### 4.1 Distinct *hTERT* promoter DNA methylation patterns identified in APL cell lines and patients

Driving mutations in *hTERT* promoter have been found in over 50 cancer types (Vinagre *et al.*, 2013). Although these mutations are frequent in certain types of cancers, they are rarely found in other types, including breast, prostate, lung cancers, and leukemia. In the absence of *hTERT* mutations, the sustained expression and overexpression of *hTERT* in cancer cells may occur through an epigenetic switch. It can be hypothesized that this switch mechanism mimics the consequences of promoter mutations. It has been well documented that DNA methylation, histone acetylation, and methylation are involved in the regulation of *hTERT* transcription (Leao *et al.*, 2018; Yuan and Xu, 2019). However, the role and the molecular mechanisms are not well depicted and can be even contradictory due to the different cellular models, the different areas within the *hTERT* promoter studied, and the various methods used to analyze these epigenetic modifications. Furthermore, understanding the epigenetic regulation of *hTERT* requires a global analysis of the relationship between DNA methylation, histone modifications, and nucleosome positioning.

Our previous study performed on APL cell lines identified two distinct functional domains in *hTERT* promoter, one distal and one proximal, differentially methylated (Azouz *et al.*, 2010). These results are in agreement with the recent identification of the *TERT* hypermethylation oncologic region, a 433‐bp genomic region located −159 to −591 bp upstream the TSS (Castelo‐Branco *et al.*, 2016; Leao *et al.*, 2019; Lee *et al.*, 2019), as a potential biomarker in several cancers. This region is of major importance, because its methylation status correlates with *hTERT* expression. A similar profile has also been reported in other cancer cells expressing telomerase (Zinn *et al.*, 2007). In the present study, we depicted similar functional regions of the *hTERT* core promoter in APL patients at diagnosis. Upon disease remission, all APL patients present a decreased DNA methylation in the distal domain of the *hTERT* core promoter, as compared to patient at diagnosis, leading to a pattern of methylation similar to that in healthy individuals. *In vivo*, ATRA treatment causes the abnormal promyelocytes to differentiate into mature leukocytes; however, their clearance is not known. The results obtained in patients favor the hypothesis that the difference in the methylation pattern at the *hTERT* core promoter between patients at diagnosis and patients at remission after ATRA treatment is not due to a change of pattern during differentiation of abnormal promyelocytes but rather to a disappearance of predominant abnormal promyelocytes in favor of normal promyelocytes. This observation on patient specimen is in perfect agreement with the results obtained in NB4‐LR1 cells showing that long‐term ATRA treatment induced a decrease of DNA methylation in the same region, which is accompanied by *hTERT* repression.

Altogether, this finding, however, reinforces the idea that DNA methylation pattern of this region represents a potential indicator for monitoring the disease outcome. Furthermore, it raises the question of whether targeting the methylation status of this distal region of *hTERT* core promoter may have a therapeutic interest.

We used nucleosome occupancy and methylation sequencing (NOMe‐seq) assay for the first time to measure simultaneously endogenous DNA methylation and nucleosome occupancy at *hTERT* gene promoter. The low level of methylation at *hTERT* TSS was similar between cells that repressed *hTERT* (ATRA‐treated NB4‐LR1 and ATRA + ATO‐treated NB4‐LR1^SFD^ cells) and cells that maintained *hTERT* expression (untreated NB4‐LR1 and NB4‐LR1^SFD^ cells, ATRA‐ or ATO‐treated NB4‐LR1^SFD^ cells). Despite the constant low level of methylation in the proximal part of *hTERT* promoter, modifications in chromatin accessibility were observed correlated with a reduction of active histone marks and *hTERT* transcriptional activity. Interestingly, our study shows that some sites of differential accessibility did not show the expected differential DNA methylation pattern indicating that, as already reported (Collings and Anderson, 2017), some epigenomic chromatin components other than DNA methylation, including histone modifications, are responsible for differential chromatin accessibility.

### 4.2 Identification of specific histone modification marks associated with *hTERT* regulation by retinoids

We showed in NB4‐LR1 cells that *hTERT* promoter has the features of a bivalent promoter, that is, the simultaneous presence of the repressive mark H3K27Me3 and the activation mark H3K4Me3 around the TSS. These marks are commonly seen on bivalent genes ‘poised’ for activation upon stem cell commitment and differentiation. In embryonic stem cells, bivalent promoters may achieve the possibility to respond rapidly to incoming signals by suppressing the formation of active RNA polymerase II complexes on the one hand, and, on the other hand, not allow other less reversible suppressive regulatory mechanisms, like DNA methylation, to silence genes. This feature demonstrates the plasticity of the *hTERT* gene in NB4‐LR1 cell variants contributing to its reactivation during cellular transformation and tumorigenesis in a permissive environment. Indeed, ATRA treatment induced a strong decrease of the active histone marks, whereas enrichment of repressive marks was observed. By contrast, in NB4‐LR1^SFD^ cells, the levels of H3K27Me3 were significantly lower. In this cell line, only the ATRA and ATO combined treatment induced a slight enrichment of this repressive mark associated with a decrease of the active histone marks more important for H3Ac than H3K4Me3.

Importantly, this study points to a role of a new region in activating *hTERT*. This region is localized outside the minimal promoter approximately 5 kb upstream of the TSS and has been previously described as an enhancer (Eldholm *et al.*, 2014). This domain is differentially methylated in both cell lines, being hypermethylated in NB4‐LR1 cells and hypomethylated in NB4‐LR1^SFD^ cells. Interestingly and in contrast with NB4‐LR1 cells, in NB4‐LR1^SFD^ cells, this region of *hTERT* promoter was enriched in both H3Ac and H3K4Me3 active marks. This feature has also been observed in one of the APL patient samples analyzed in this study. H3K4Me3 is a predominant feature of active promoters, but detectable levels of this modification are also observed at active enhancers (Pekowska *et al.*, 2011). Compared to NB4‐LR1 cells, a high level of H3K4Me3 was observed in the enhancer domain of *hTERT* in the untreated NB4‐LR1^SFD^ cells. Although the mechanisms underlying this difference in H3K4Me3 deposition at *hTERT* enhancer remain to be further explored, one potential explanation is that it may reflect a difference in the DNA methylation pattern of this region. Indeed, trimethylation of H3K4 (H3K4Me3) is mostly performed by the CxxC finger protein 1 (Cfp1), a subunit of the human Set1 complex, which influence chromatin structure through its binding to unmethylated CpGs and links H3K4Me3 with CpG islands (Lee and Skalnik, 2005; Thomson *et al.*, 2010). However, Cpf1 also plays a role in the production of H3K4Me3 at other regulatory regions, including distal enhancers (Clouaire *et al.*, 2012; van de Lagemaat *et al.*, 2018). As in NB4‐LR1^SFD^ cells, the *hTERT* enhancer region is mainly hypomethylated compared to NB4‐LR1 cells, it could be targeted by Cfp1 (or other CpG‐binding proteins) leading to the ectopic deposition of H3K4Me3 and to an aberrantly elevated level of transcription both from the enhancer and from the nearby promoter. This feature can explain the higher constitutive expression of *hTERT* in NB4‐LR1^SFD^ cells than in NB4‐LR1 cells. In contrast in NB4‐LR1 cells, densely methylated CpGs in the *hTERT* enhancer region are likely to attract methyl‐CpG‐binding proteins that recruit enzymes reinforcing repressive histone modifications (i.e., H3K27Me3).

**Fig. 6:**
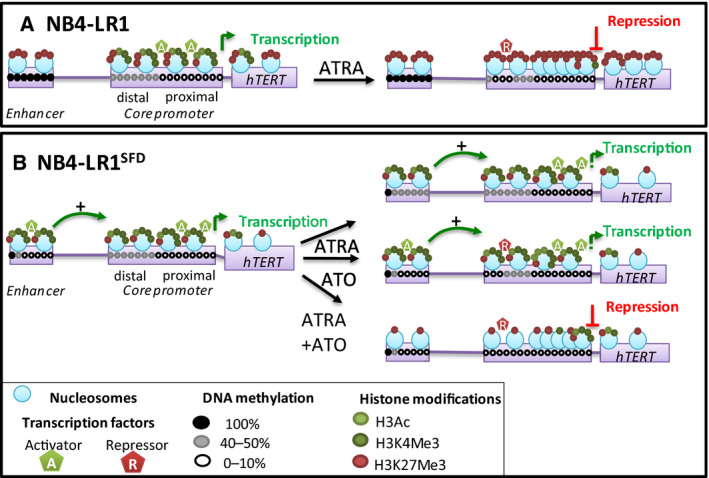
Proposed model of epigenetic regulation of *hTERT* expression. *hTERT* promoter and enhancer exhibit distinct epigenetic features in NB4‐LR1 (A) and NB4‐LR1^SFD^ (B) cells. (A) In NB4‐LR1 cells, under ATRA treatment, the repressive histone mark (H3K27Me3) is enriched at the *hTERT* core promoter whereas the active marks (H3Ac and H3K4Me3) are depleted. These modifications are associated with an increase in the number of nucleosomes, a decrease in chromatin accessibility, and *hTERT* repression. In addition, the DNA methylation of the distal part of *hTERT* core promoter decreases. This hypomethylation pattern would possibly allow the binding of a repressive factor (R) partly responsible for ATRA‐induced *hTERT* repression. The enhancer domain of hTERT remains unmodified. (B) In NB4‐LR1^SFD^ cells, the enhancer domain is characterized by active histone marks and hypomethylated DNA. This decrease would possibly allow the binding of an activator (A). *hTERT* is repressed only after treatment with ATRA and ATO in combination. This *hTERT* repression is associated, as in NB4‐LR1 cells, with a demethylation of the distal *hTERT* promoter and a condensation of the chromatin in the proximal part of *hTERT* promoter. Moreover, in contrast with NB4‐LR1, in NB4‐LR1^SFD^ cells, *hTERT* repression involves the inactivation of the enhancer region of *hTERT* characterized by a decrease of the histone active marks. ATRA treatment alone does not induce any modification of DNA methylation, histone modifications, and chromatin accessibility in the *hTERT* core promoter. However, a weak but significant increase of DNA methylation in the enhancer region could partly explain the initial *hTERT* repression observed after ATRA treatment of the NB4‐LR1^SFD^ cells. ATO treatment alone does not change DNA methylation of this enhancer region. However, it decreases the DNA methylation of the distal *hTERT* promoter. As mentioned above for NB4‐LR1 cells, this can favor the binding of a repressor and explain partly the initial repression of *hTERT* observed after ATO treatment. From this model we extrapolate that an inhibition of the enhancer activity associated with epigenetic modifications at both distal and proximal domain of *hTERT* promoter are necessary for a full repression of *hTERT* promoter.

In NB4‐LR1^SFD^ cells, only a combination of ATRA and ATO treatments induced *hTERT* repression associated with a drastic drop of the levels of H3K4Me3 and H3Ac marks at *hTERT* enhancer and promoter. In NB4‐LR1 cells, the *hTERT* enhancer is not active and consequently ATRA alone was able to induce *hTERT* repression provided both the enhancer and promoter region of *hTERT* have been enriched with the H3K27Me3 repressive mark and *hTERT* promoter depleted of the and H3K4Me3/H3Ac active marks (Fig. 6).

Altogether, these results suggest that the epigenetic features of this region could play a major role and dictate the context‐dependent *hTERT* transcriptional outcome, through differential recruitment of transcription factors and other chromatin‐modifying enzymes. *In silico* analysis of this region identifies putative consensus binding sites for multiple interacting transcription factors, including RARα/RXRγ, PPARα/RXRα, Sp1, and GABPα. Factors, such as CTCF, that interact with *hTERT* promoter (either proximal promoter or enhancer) are known for organizing global chromosomal architecture, changing accessibility, but also possible interactions far away in distance. Thus, it can be proposed that a potential cross‐talk exists between the enhancer and the core promoter of *hTERT* gene to orchestrate its expression. As transcription factors could interact with some epigenetic modifiers, a better understanding of the cooperation between transcription factors and the epigenetic landscape will offer hope to develop new drug opportunities. The epigenetic modifications reported above could partially participate in regulation of *hTERT* gene expression via telomere looping in a context not related to telomere length modifications as suggested in the present study and also recently reported (Kim and Shay, 2018).

Retinoid‐based therapies have low systemic toxicity when compared to conventional chemotherapeutics. Recent studies indicate that ATRA‐based therapy may be of benefit to patients with other cancers. However, the individuality in the clinical response shows clearly that a treatment protocol may not be effective in all cases. The present work identifies some specific features of *hTERT* epigenetic landscape that could be used to predict the response of patients to ATRA‐based therapy.

## 5 Conclusions

Together, our results suggest that the local chromatin accessibility of the core promoter of *hTERT* gene is likely the most important feature controlling the transcriptional expression of *hTERT* gene. However, it could not be excluded that the methylation pattern of the enhancer domain of *hTERT* gene is associated with specific histone modifications that could play a role in *hTERT* activation.

Therefore, our results suggest a very complex relationship between the epigenetic state of *hTERT* promoter and transcriptional activity and, thereby, force the revisiting of some previously proposed concepts regarding *hTERT* regulation. The analysis of the epigenetic status of *hTERT* as performed in this study can provide the basis for further works to extend these findings and translate them into promising new approaches for the treatment of a broad range of cancers. As next generation sequencing technologies associated with new bioinformatics techniques and analysis tools are rapidly evolving, new opportunities are provided to identify epigenetic landscape changes that can be successfully and reliably used in clinical practice to follow the disease and the response to treatment.

## Conflict of interest

The authors declare no conflict of interest.

## Author contributions

DG, CB, and EN performed the experimental work. RK performed the APL patient dataset analysis. DG, EN, RK, and ES‐B analyzed the data. MP‐C and EC contributed biological materials. DG, CB, EN, MP‐C, EC, and PF contributed to the drafted manuscript and to its critical revision. ES‐B designed the study and wrote the manuscript. The final manuscript was read and approved by all authors. All authors discussed the results and commented on the manuscript.

## Supporting information


**Fig. S1.** Schematic representation of *hTERT* gene.

**Fig. S2.** Localization of the probes used in the FISH assay.

**Fig. S3.** Nucleosome occupancy and endogenous CpG methylation at the *hTERT* gene promoter.

**Fig. S4.** Genome browser screenshot of two high‐risk (pz‐284 and pz‐289) and one primary (pz‐302) APL patient samples ChIP‐seq results at the *hTERT* locus before and after *ex vivo* ATRA treatment for 24 h.

**Table S1.** Primers sequences (5′ to 3′) and amplification conditions used for NOMe‐seq, ChIP, and *hTERT* expression and promoter gene analysis.
**Table S2.** List of single nucleotide polymorphisms (SNPs) identified by genetic sequencing of the hTERT promoter.

## Data Availability

The APL dataset analyzed in the current study is available at the Gene Expression Omnibus (https://www.ncbi.nlm.nih.gov/geo/query/acc.cgi?acc=GSE42119) and at the BLUEPRINT data portal (http://experiment://dcc.blueprint-epigenome.eu/#/experiment).
