# Supplementary figures and images for: Complex context relationships between DNA methylation and accessibility, histone marks, and hTERT gene expression in acute promyelocytic leukemia cells: perspectives for all‐trans retinoic acid in cancer therapy
Source: Mol Oncol. 2020 Apr 22;14(6):1310–26. doi: 10.1002/1878-0261.12681 (PMC7266276; doi:10.1002/1878-0261.12681)

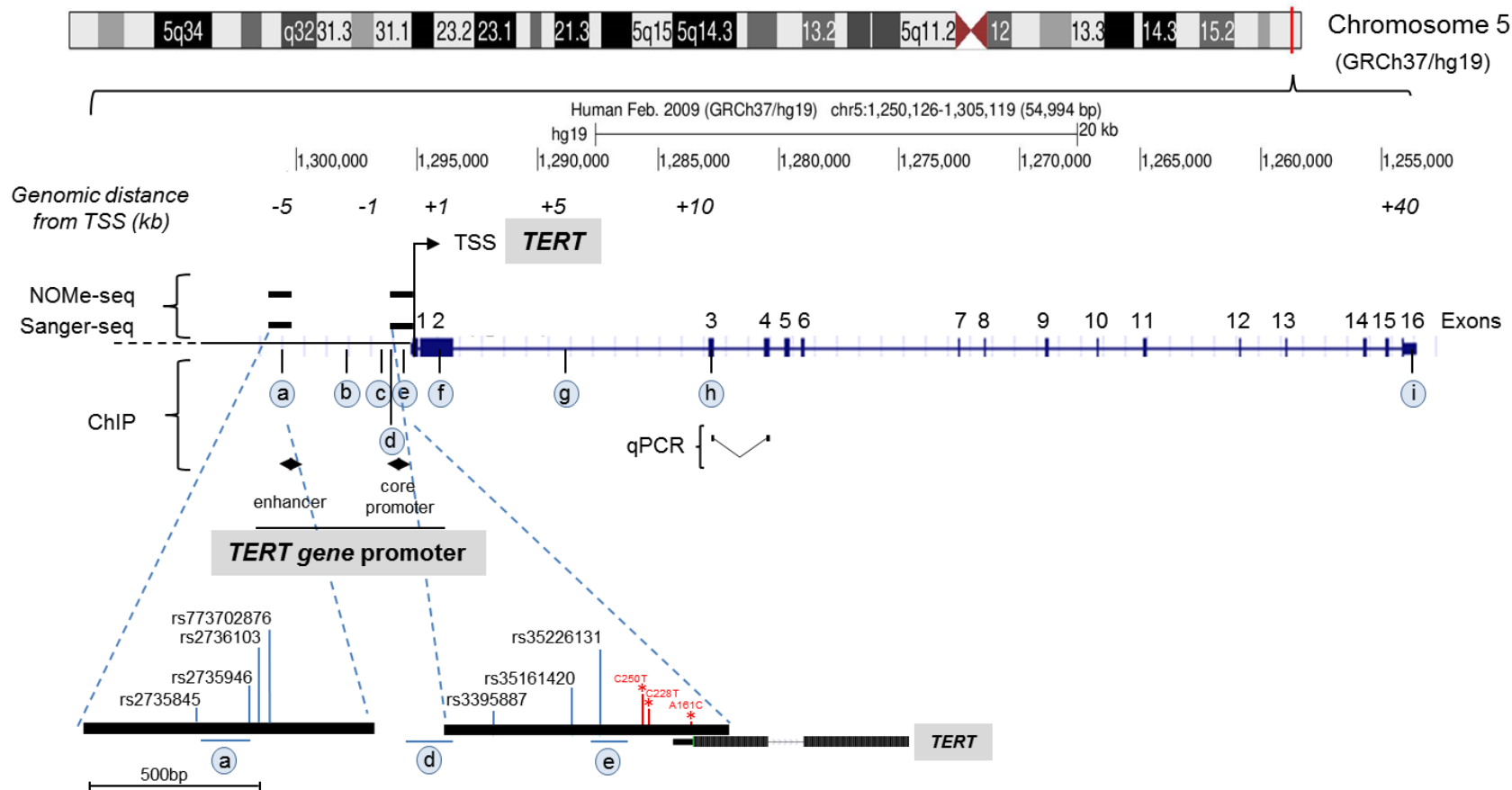

Supplement: Supplementary file 1 — Fig. S1. Schematic representation of hTERT gene. [file MOL2-14-1310-s001.pdf]

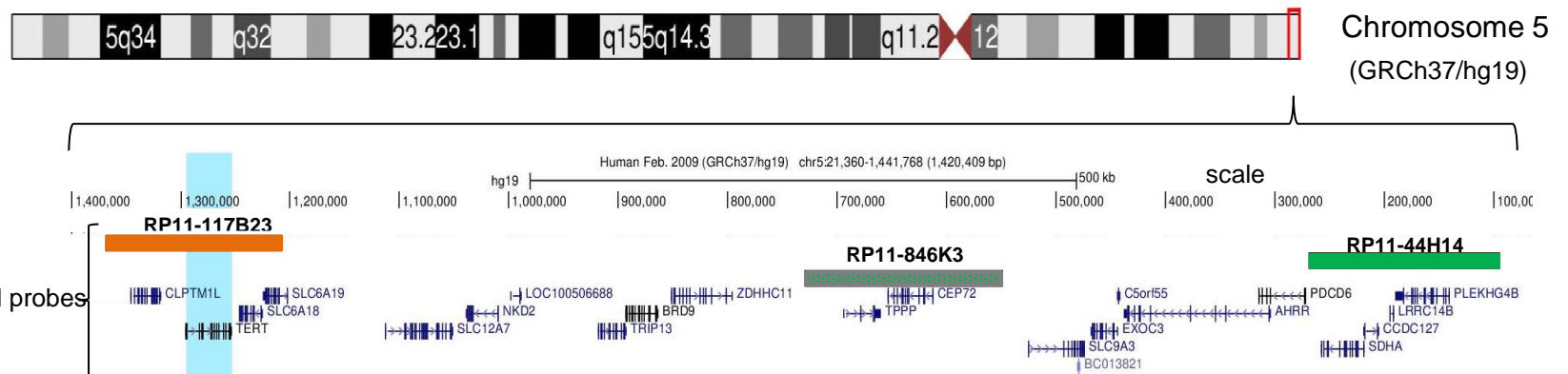

Supplement: Supplementary file 2 — Fig. S2. Localization of the probes used in the FISH assay. [file MOL2-14-1310-s002.pdf]

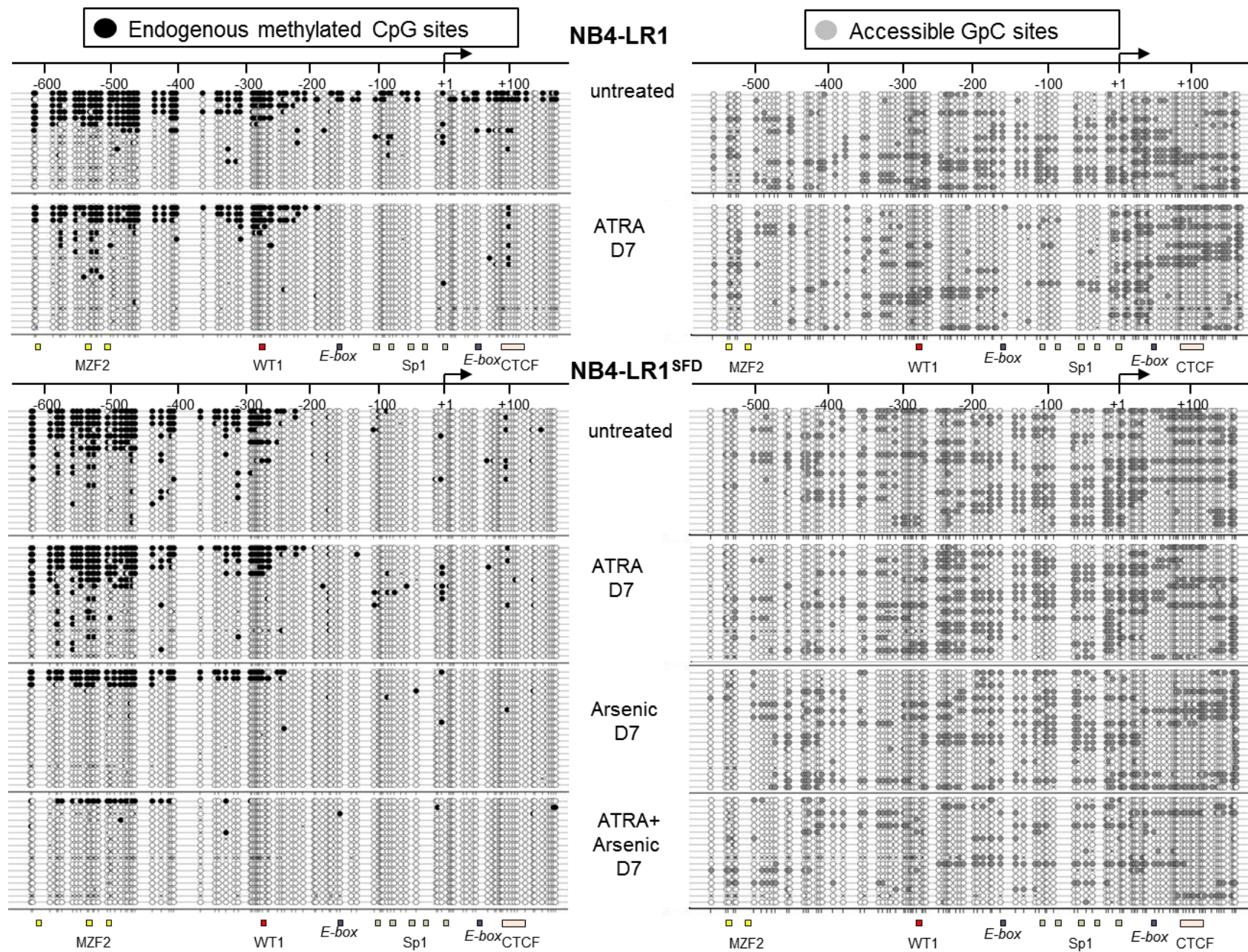

Supplement: Supplementary file 3 — Fig. S3. Nucleosome occupancy and endogenous CpG methylation at the hTERT gene promoter. [file MOL2-14-1310-s003.pdf]

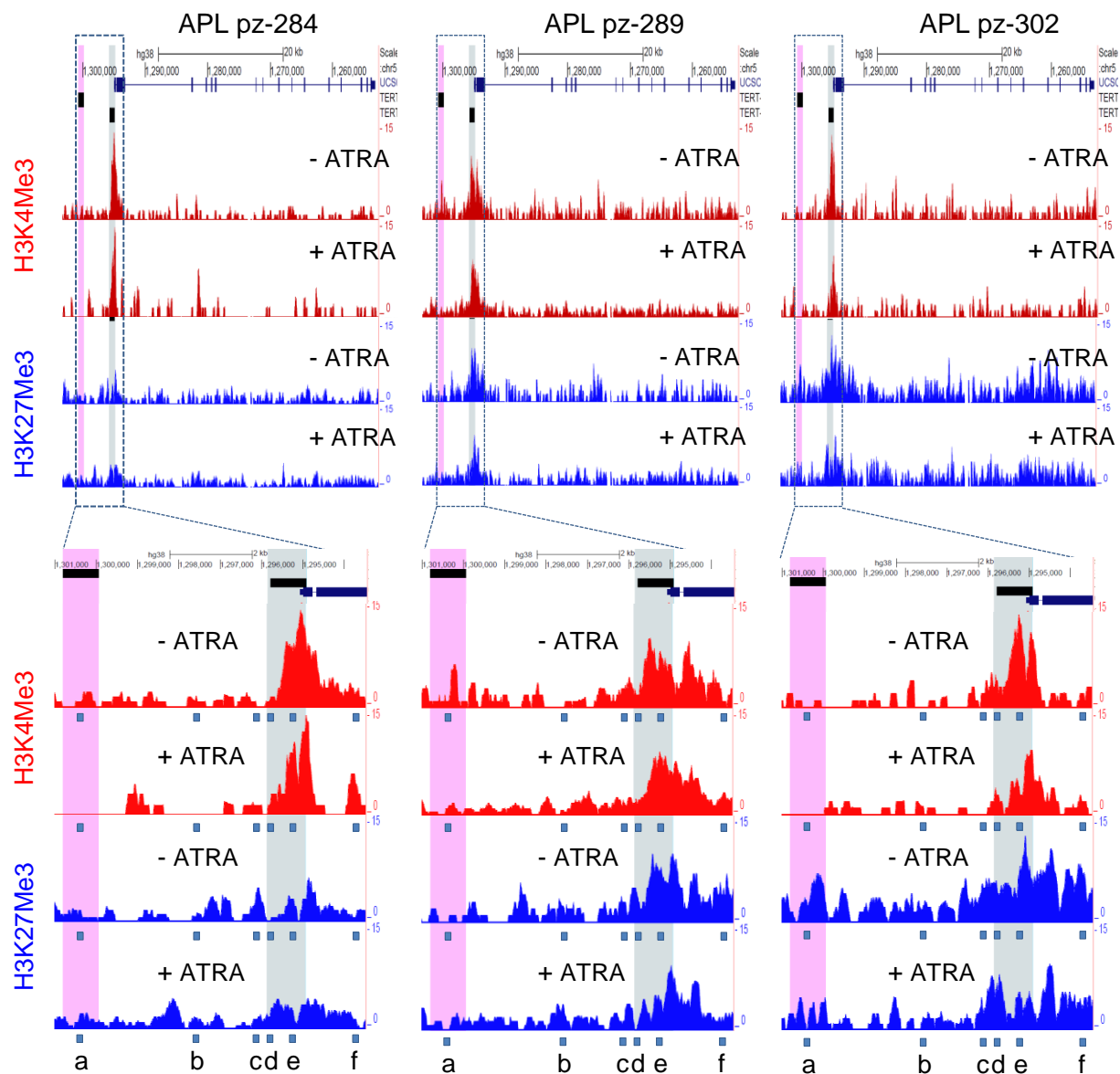

Supplement: Supplementary file 4 — Fig. S4. Genome browser screenshot of two high‐risk (pz‐284 and pz‐289) and one primary (pz‐302) APL patient samples ChIP‐seq results at the hTERT locus before and after ex vivo ATRA treatment for 24 h. [file MOL2-14-1310-s004.pdf]
