# Supplementary material for: Complex context relationships between DNA methylation and accessibility, histone marks, and hTERT gene expression in acute promyelocytic leukemia cells: perspectives for all‐trans retinoic acid in cancer therapy
Source: Mol Oncol. 2020 Apr 22;14(6):1310–26. doi: 10.1002/1878-0261.12681 (PMC7266276; doi:10.1002/1878-0261.12681)
Supplement: Supplementary file 5 — Table S1. Primers sequences (5′ to 3′) and amplification conditions used for NOMe‐seq, ChIP, and hTERT expression and promoter gene analysis. Table S2. List of single nucleotide polymorphisms (SNPs) identified by genetic sequencing of the hTERT promoter. [file MOL2-14-1310-s005.pdf]

Table S1: Primers sequences (5' to 3') and amplification conditions used for NoMe-seq, ChIP and *hTERT* expression and promoter gene analysis. The positions of the amplicons are indicated in **Fig S1**.

Primer name	Primers*	Annealing °C
	**Sequencing**	
hTERT-5kbpromoter-seq	FW : ACCCTTCTCAAGGGAAAACCAGA<br>RV : TGGAATCATTCAATCCTTGGGG	60
hTERT-promoter-seq	FW (-712) : AACAGATTTGGGGTGGTTTG<br>RV (-435) : CTGGCCTGATCCGGAGAC	54-56
	FW (-514) : TCCCCTTCACGTCCGGCAT<br>RV (+53) : TCCCACGTGCGCAGCAGGAC	60
	FW (-347) : GGCCGATTCGACCTCTCT<br>RV (+120) : AGCACCTCGCGGTAGTGG	60
	**NOMEseq**	
hTERT-5kbpromoter-NOMeSeq	FW-5kbBS3F : TTTGGAGAGAGGAGTTTGAG<br>RV-5kbBS2R : TCATTCAATCCTTAAAAATAAAAATAAATA	51
hTERT-promoter-NOMeSeq :	FW BS1F : GGGYTTGTGTTAAGGAGYTTAAGT<br>RV BS3R : CCARCCCTAAARCCCCAA	58
	**PCR**	
hTERT-qPCR	FW : CGGAAGAGTGTCTGGAGCAA<br>RV : CTCCCACGACGTAGTCCATG	58
GAPDH-qPCR	FW : CACCCATGGCAAATTCCATGGC	58

	RV : GCATTGCTGATGATCTTGAGGCT	
**ChIP**		
hTERT-ChIP-up5kb (-5401)	FW : CCAAAGGCGTAAAACAGGAA<br>RV : CCTCGTGTACTTTCCCTTGC	60
hTERT-ChIP-up2.5kb (-2582)	FW : AAACTTCCCTGGGCTCAAGT<br>RV : CGGTGTATCCCCAGTCTACG	60
hTERT-ChIP-up1.0kb (-1102)	FW : GTTTCTCGCCCCTTAGATCC<br>RV : GCAGGACAGCTGAGGACTTC	60
hTERT-ChIP-up0.8kb (-773)	FW : CTCCATTTCCCACCCTTTCT<br>RV : ACTTGGGCTCCTTGACACAG	60
hTERT-ChIP-up0.2kb (-233)	FW : CAGGCCGGGCTCCCAGTGGA<br>RV : GGAAGGTGAAGGGGCAGGAC	65
hTERT-ChIP-down1.3kb (+1310)	FW : TGCCCCAGCGCTACTGGCAAA<br>RV : TCGCAGCGGGCAGTGCGTCTTGA	67
hTERT-ChIP-down6.5kb	FW : TGGCAACGCTTGTCACCTTA<br>RV : ACGTCAATCCATGTGAGGGG	60
hTERT-ChIP-down12.5kb	FW : CGTCTTTCTTTTATGTCACGGAG<br>RV : AATGCTTTGCAACTTGCTCCA	60
hTERT-ChIP-down41.7kb	FW : CCATCCCCAGATTCGCCATT<br>RV : CTGTGTACAGGGCACACCTT	60

*Y = C or T and R = A or G

Table S2: List of single nucleotide polymorphisms (SNPs) identified by genetic sequencing of the hTERT promoter.

SNPs	Allele	References
rs33958877<br>GRCh38.p12-g.1295567<br>GRCH37.p13-g.1295682	A/C (NB4-LR1 and NB4-LR1^{SFD})	(Montesanto et al., 2018)
rs35161420<br>GRCh38.p12-g.1295337<br>GRCH37.p13-g.1295452	G/C (NB4-LR1 and NB4-LR1^{SFD})	(Montesanto et al., 2018)
rs35226131<br>GRCh38.p12-g.1295258<br>GRCH37.p13-g.1295373	A/G (NB4-LR1 and NB4-LR1^{SFD})	(Montesanto et al., 2018)<br>(Zhang et al., 2016a)
rs2735845<br>GRCh38.p12-g.1300469<br>GRCH37.p13-g.1300584	C/G (NB4-LR1 and NB4-LR1^{SFD})	(Zhang et al., 2016b; Zhang et al., 2014)<br>(Pande et al., 2011)<br>(Beesley et al., 2011)<br>(Ge et al., 2016)<br>(Zhou et al., 2016)
rs27355946<br>GRCh38.p12-g.1300314<br>GRCH37.p13-g.1300429	A>C (NB4-LR1 and NB4-LR1^{SFD})	(Pande et al., 2011)
rs2736103<br>GRCh38.p12-g.1300286<br>GRCH37.p13-g.0300401	A>G (NB4-LR1 and NB4-LR1^{SFD})	(Pande et al., 2011)
rs773702876<br>GRCh38.p12-g.1300251_1300255<br>GRCH37.p13-g.1300366_1300370	delTGCCT (NB4-LR1 and NB4-LR1^{SFD})	-

Supplementary references

Beesley, J, Pickett, HA, Johnatty, SE, Dunning, AM, Chen, X, Li, J, Michailidou, K, Lu, Y, Rider, DN, Palmieri, RT, et al. (2011) Functional polymorphisms in the TERT promoter are associated with risk of serous epithelial ovarian and breast cancers. *PLoS One* **6**, e24987.

Ge, M, Shi, M, An, C, Yang, W, Nie, X, Zhang, J, Lv, Z, Li, J, Zhou, L, Du, Z, et al. (2016) Functional evaluation of TERT-CLPTM1L genetic variants associated with susceptibility of papillary thyroid carcinoma. *Scientific reports* **6**, 26037.

Montesanto, A, Bonfigli, AR, Crocco, P, Garagnani, P, De Luca, M, Boemi, M, Marasco, E, Pirazzini, C, Giuliani, C, Franceschi, C, et al. (2018) Genes associated with Type 2 Diabetes and vascular complications. *Aging (Albany NY)* **10**, 178-196.

Pande, M, Spitz, MR, Wu, X, Gorlov, IP, Chen, WV and Amos, CI (2011) Novel genetic variants in the chromosome 5p15.33 region associate with lung cancer risk. *Carcinogenesis* **32**, 1493-1499.

Zhang, M, Wang, Z, Obazee, O, Jia, J, Childs, EJ, Hoskins, J, Figlioli, G, Mocci, E, Collins, I, Chung, CC, et al. (2016a) Three new pancreatic cancer susceptibility signals identified on chromosomes 1q32.1, 5p15.33 and 8q24.21. *Oncotarget* **7**, 66328-66343.

Zhang, Y, Zhang, X, Zhang, H, Zhai, Y, Wang, Z, Li, P, Yu, L, Xia, X, Zhang, Y, Zeng, Y, et al. (2016b) Common variations in TERT-CLPTM1L locus are reproducibly associated with the risk of nasopharyngeal carcinoma in Chinese populations. *Oncotarget* **7**, 759-770.

Zhang, Y, Zhao, M, Shen, L, Ren, Y, Su, L, Li, X, Yin, Z and Zhou, B (2014) Genetic polymorphisms of TERT and CLPTM1L and risk of lung cancer: a case-control study in northeast Chinese male population. *Medical oncology* **31**, 18.

Zhou, L, Fu, G, Wei, J, Shi, J, Pan, W, Ren, Y, Xiong, X, Xia, J, Shen, Y, Li, H, et al. (2016) The identification of two regulatory ESCC susceptibility genetic variants in the TERT-CLPTM1L loci. *Oncotarget* **7**, 5495-5506.
