# Supplementary material for: Complex context relationships between DNA methylation and accessibility, histone marks, and hTERT gene expression in acute promyelocytic leukemia cells: perspectives for all‐trans retinoic acid in cancer therapy
Source: Mol Oncol. 2020 Apr 22;14(6):1310–26. doi: 10.1002/1878-0261.12681 (PMC7266276; doi:10.1002/1878-0261.12681)
Supplement: Supplementary file 6 [file MOL2-14-1310-s006.pdf]

Complex context-relationships between DNA methylation and accessibility, histone marks, and *hTERT* gene expression in acute promyelocytic leukemia cells: perspective for all-*trans* retinoic acid (ATRA) in cancer therapy.

Delphine Garsuault[1,2,3], Claire Bouyer[1,2], Eric Nguyen[1,2], Rohan Kandhari[1,4] Martina Prochazkova-Carlotti[5], Edith Chevret[5], Patricia Forgez[1,2], Evelyne Ségal-Bendirdjian[1,2]

[1] INSERM UMR-S 1124, Team: Cellular Homeostasis, Cancer, and Therapies Paris, France

[2] Paris-Descartes University, Paris Sorbonne Cité, Paris, France

[3] Paris-Sud University, Paris-Saclay University, Orsay, France

[4] Indian Institute of Technology BHU, Varanasi, India

[5] INSERM U1053, Team Cutaneous Lymphoma Oncogenesis Bordeaux, France

Supporting information

Fig. S1: Schematic representation of *hTERT* gene. The figure encompasses the hg19 coordinates chr5: 1,250,126-1,305,119. The scale of this diagram is given above the gene. The overall intron/exon structure of the gene, which consists of 16 exons (blue boxes numbered from 1 to 16) and 15 introns, is represented. The transcription start site (TSS) is indicated by an arrow. The locations of enhancer and core promoter elements are indicated. The encircled letters under the schema indicate the localization on the gene where the different probe sets localize for analysis of ChIP studies. The regions sequenced in NOMe-seq and Sanger sequencing are indicated by a solid dark line above the gene. qPCR primers used for measuring *hTERT* expression are indicated. The localization of the SNP identified in NB4-LR1 and NB4-LR1^{SFD} are noted. The *hTERT* promoter mutations most frequently described in the literature are also indicated for information.

Fig. S2: Localization of the probes used in the FISH assay. The FISH probes (RP11-117B23 for hTERT locus, RP11-44H14 for subtelomeric region 5p and RP11-846K3 for the intermediary region) used in the present study are indicated. The region corresponding to the *hTERT* gene is highlighted in blue.

Fig. S3: Nucleosome occupancy and endogenous CpG methylation at the *hTERT* gene promoter. NOMe-seq was used to map the position of nucleosome on individual DNA molecules at the *hTERT* gene promoter in NB4-LR1 and NB4-LR1^{SFD} cells treated or not as indicated. Left panel

indicates endogenous DNA methylation, right panel chromatin accessibility. Each horizontal row in each panel represents the same DNA molecule. For the DNA methylation plots: vertical bars below the line: CpG sites; black filled circles: methylated CpG dinucleotides; white filled circles: unmethylated CpG dinucleotides. For the accessibility plots: vertical bar below the line, GpC sites; grey circles: methylated GpC, accessible to M.CviPI; white circles : unmethylated GpC, inaccessible to M.CviPI.

Fig. S4: Genome browser screenshot of two high-risk (pz-284 and pz-289) and one primary (pz-302) APL patient samples ChIP-seq results at the *hTERT* locus before and after *ex vivo* ATRA treatment for 24h. In red the H3K4Me3, in blue the H3K27Me3 ChIP-sequencing data. As in Fig.4, semitransparent grey and pink color labels the analyzed regions of *hTERT* proximal promoter and enhancer, respectively. The encircled letters under the schema indicate the localization on where the different probe sets localize for analysis of ChIP studies as presented in **Fig.4** and **Fig. S1**.

Table S1: Primers sequences (5' to 3') and amplification conditions used for NoMe-seq, ChIP and *hTERT* expression and promoter gene analysis. The positions of the amplicons are indicated in **Fig S1**.

**Primer name**	**Primers***	**Annealing °C**
	**Sequencing**	
hTERT-5kbpromoter-seq	FW : ACCCTTCTCAAGGGAAAACCAGA<br>RV : TGGAATCATTCAATCCTTGGGG	60
hTERT-promoter-seq	FW (-712) : AACAGATTTGGGGTGGTTTG<br>RV (-435) : CTGGCCTGATCCGGAGAC	54-56
	FW (-514) : TCCCCTTCACGTCCGGCAT<br>RV (+53) : TCCCACGTGCGCAGCAGGAC	60
	FW (-347) : GGCCGATTCGACCTCTCT<br>RV (+120) : AGCACCTCGCGGTAGTGG	60
	**NOMEseq**	
hTERT-5kbpromoter-NOMeSeq	FW-5kbBS3F : TTTGGAGAGAGGAGTTTGAG<br>RV-5kbBS2R : TCATTCAATCCTTAAAAATAAAAATAAATA	51
hTERT-promoter-NOMeSeq :	FW BS1F : GGGYTTGTGTTAAGGAGYTTAAGT<br>RV BS3R : CCARCCCTAAARCCCCAA	58
	**PCR**	
hTERT-qPCR	FW : CGGAAGAGTGTCTGGAGCAA<br>RV : CTCCCACGACGTAGTCCATG	58
GAPDH-qPCR	FW : CACCCATGGCAAATTCCATGGC	58

	RV : GCATTGCTGATGATCTTGAGGCT	
**ChIP**		
hTERT-ChIP-up5kb (-5401)	FW : CCAAAGGCGTAAAACAGGAA<br>RV : CCTCGTGTACTTTCCCTTGC	60
hTERT-ChIP-up2.5kb (-2582)	FW : AAACTTCCCTGGGCTCAAGT<br>RV : CGGTGTATCCCCAGTCTACG	60
hTERT-ChIP-up1.0kb (-1102)	FW : GTTTCTCGCCCCTTAGATCC<br>RV : GCAGGACAGCTGAGGACTTC	60
hTERT-ChIP-up0.8kb (-773)	FW : CTCCATTTCCCACCCTTTCT<br>RV : ACTTGGGCTCCTTGACACAG	60
hTERT-ChIP-up0.2kb (-233)	FW : CAGGCCGGGCTCCCAGTGGA<br>RV : GGAAGGTGAAGGGGCAGGAC	65
hTERT-ChIP-down1.3kb (+1310)	FW : TGCCCCAGCGCTACTGGCAAA<br>RV : TCGCAGCGGGCAGTGCGTCTTGA	67
hTERT-ChIP-down6.5kb	FW : TGGCAACGCTTGTCACCTTA<br>RV : ACGTCAATCCATGTGAGGGG	60
hTERT-ChIP-down12.5kb	FW : CGTCTTTCTTTTATGTCACGGAG<br>RV : AATGCTTTGCAACTTGCTCCA	60
hTERT-ChIP-down41.7kb	FW : CCATCCCCAGATTCGCCATT<br>RV : CTGTGTACAGGGCACACCTT	60

*Y = C or T and R = A or G

Table S2: List of single nucleotide polymorphisms (SNPs) identified by genetic sequencing of the hTERT promoter.

SNPs	Allele	References
rs33958877<br>GRCh38.p12-g.1295567<br>GRCH37.p13-g.1295682	A/C (NB4-LR1 and NB4-LR1^{SFD})	(Montesanto et al., 2018)
rs35161420<br>GRCh38.p12-g.1295337<br>GRCH37.p13-g.1295452	G/C (NB4-LR1 and NB4-LR1^{SFD})	(Montesanto et al., 2018)
rs35226131<br>GRCh38.p12-g.1295258<br>GRCH37.p13-g.1295373	A/G (NB4-LR1 and NB4-LR1^{SFD})	(Montesanto et al., 2018)<br>(Zhang et al., 2016a)
rs2735845<br>GRCh38.p12-g.1300469<br>GRCH37.p13-g.1300584	C/G (NB4-LR1 and NB4-LR1^{SFD})	(Zhang et al., 2016b; Zhang et al., 2014)<br>(Pande et al., 2011)<br>(Beesley et al., 2011)<br>(Ge et al., 2016)<br>(Zhou et al., 2016)
rs27355946<br>GRCh38.p12-g.1300314<br>GRCH37.p13-g.1300429	A>C (NB4-LR1 and NB4-LR1^{SFD})	(Pande et al., 2011)
rs2736103<br>GRCh38.p12-g.1300286<br>GRCH37.p13-g.0300401	A>G (NB4-LR1 and NB4-LR1^{SFD})	(Pande et al., 2011)
rs773702876<br>GRCh38.p12-g.1300251_1300255<br>GRCH37.p13-g.1300366_1300370	delTGCCT (NB4-LR1 and NB4-LR1^{SFD})	-

Supplementary references

Beesley, J, Pickett, HA, Johnatty, SE, Dunning, AM, Chen, X, Li, J, Michailidou, K, Lu, Y, Rider, DN, Palmieri, RT, et al. (2011) Functional polymorphisms in the TERT promoter are associated with risk of serous epithelial ovarian and breast cancers. *PLoS One* **6**, e24987.

Ge, M, Shi, M, An, C, Yang, W, Nie, X, Zhang, J, Lv, Z, Li, J, Zhou, L, Du, Z, et al. (2016) Functional evaluation of TERT-CLPTM1L genetic variants associated with susceptibility of papillary thyroid carcinoma. *Scientific reports* **6**, 26037.

Montesanto, A, Bonfigli, AR, Crocco, P, Garagnani, P, De Luca, M, Boemi, M, Marasco, E, Pirazzini, C, Giuliani, C, Franceschi, C, et al. (2018) Genes associated with Type 2 Diabetes and vascular complications. *Aging (Albany NY)* **10**, 178-196.

Pande, M, Spitz, MR, Wu, X, Gorlov, IP, Chen, WV and Amos, CI (2011) Novel genetic variants in the chromosome 5p15.33 region associate with lung cancer risk. *Carcinogenesis* **32**, 1493-1499.

Zhang, M, Wang, Z, Obazee, O, Jia, J, Childs, EJ, Hoskins, J, Figlioli, G, Mocci, E, Collins, I, Chung, CC, et al. (2016a) Three new pancreatic cancer susceptibility signals identified on chromosomes 1q32.1, 5p15.33 and 8q24.21. *Oncotarget* **7**, 66328-66343.

Zhang, Y, Zhang, X, Zhang, H, Zhai, Y, Wang, Z, Li, P, Yu, L, Xia, X, Zhang, Y, Zeng, Y, et al. (2016b) Common variations in TERT-CLPTM1L locus are reproducibly associated with the risk of nasopharyngeal carcinoma in Chinese populations. *Oncotarget* **7**, 759-770.

Zhang, Y, Zhao, M, Shen, L, Ren, Y, Su, L, Li, X, Yin, Z and Zhou, B (2014) Genetic polymorphisms of TERT and CLPTM1L and risk of lung cancer: a case-control study in northeast Chinese male population. *Medical oncology* **31**, 18.

Zhou, L, Fu, G, Wei, J, Shi, J, Pan, W, Ren, Y, Xiong, X, Xia, J, Shen, Y, Li, H, et al. (2016) The identification of two regulatory ESCC susceptibility genetic variants in the TERT-CLPTM1L loci. *Oncotarget* **7**, 5495-5506.
